# Potential profiles and associated factors of medication safety competence at home: a study in the older adults following percutaneous coronary intervention

**DOI:** 10.3389/fpubh.2026.1825652

**Published:** 2026-06-12

**Authors:** Furong Jiang, Yiyan Wang, Baixia Chen, Ping Dai

**Affiliations:** Department of Cardiovascular Medicine, People's Hospital of Deyang City, Deyang, Sichuan, China

**Keywords:** older adults, home medication, latent profile analysis, medication safety competence, percutaneous coronary intervention

## Abstract

**Objective:**

To explore the latent profiles of home medication safety competence in older adults patients after percutaneous coronary intervention (PCI) and analyze the associated factors of different latent profiles, so as to provide a reference for constructing precise medication management plans.

**Methods:**

Convenience sampling was used to select older adults patients who underwent PCI in the Department of Cardiology of a tertiary general hospital in Sichuan Province from February to June 2025 as the study subjects. General information questionnaire, Medication Safety Competence Scale, Short Health Literacy Scale, and Family APGAR Scale were used for investigation. Latent profile analysis was used to identify profiles of medication safety competence. We compared 1 to 4 profiles and chose the best model based on fit indices [Akaike information criterion (AIC), Bayesian information criterion (BIC), adjusted Bayesian information criterion (aBIC)], entropy, and interpretability. Multivariate logistic regression was used to analyze the associated factors.

**Results:**

A total of 393 older adults patients after PCI were included, with an average score of home medication safety competence of (102.081 ± 6.11) points. A 3-profile model was selected as the best solution (AIC = 8,837.83, BIC = 8,909.36, entropy = 0.875). Three profiles were identified: low competence group (31.0%), moderate competence group (56.0%), and high competence group (13.0%). Education level, age, health literacy, family support, and family medication reminder frequency were the main factors associated with patients' belonging to different competence profiles (all *P* < 0.05).

**Conclusion:**

There is significant population heterogeneity in home medication safety competence among older adults patients after PCI. In nursing practice, stratified and precise intervention strategies should be adopted based on the characteristics and associated factors of different competence profiles. Special attention should be paid to patients with low education level, advanced age, low health literacy, and weak family support. Enhancing patients' health literacy and strengthening the family support system are essential to promote and maintain their home medication safety competence.

## 1 Introduction

Coronary atherosclerotic heart disease (CHD), commonly known as coronary heart disease, is a cardiac condition caused by the narrowing or blockage of coronary arteries due to atherosclerosis. This leads to myocardial ischemia, hypoxia, and even necrosis (1). Cardiovascular diseases remain the leading cause of death globally. In 2021, the number of cases reached 254.28 million worldwide, highlighting their high incidence and severe impact (2). With the accelerating aging of the population, the prevalence of CHD among people aged 60 and older is as high as 2,943 per 100,000. The disease not only severely affects patients' quality of life and physiological function but also imposes a substantial burden on families and society (3). Percutaneous coronary intervention (PCI) has become one of the most effective treatments for CHD, owing to its advantages such as minimal invasiveness and low complication rates (4). However, long-term standardized medication is required after PCI to control risk factors and prevent restenosis and recurrence (5). Older adults CHD patients often have comorbidities such as hypertension and diabetes. The rate of polypharmacy in this group is as high as 70.8%, resulting in high medication complexity (6). During long-term home-based medication therapy, these patients commonly face challenges including poor medication adherence, difficulty in recognizing adverse drug reactions, and insufficient self-management skills. These issues significantly compromise treatment efficacy and health outcomes (7). Therefore, improving home medication safety competence in older adults patients after PCI is crucial.

Medication safety competence in the older adults refers to the comprehensive ability of older patients to use medications correctly, consistently, and appropriately at home. This ability is built upon essential medication knowledge, positive beliefs about treatment, and effective self-management behaviors, supported by family and social systems, enabling the timely identification and handling of medication-related issues (8). It encompasses a broad range of components, including understanding basic drug information, therapeutic purposes, and potential adverse reactions, as well as practicing medication adherence, self-monitoring, health self-assessment, effective communication with healthcare providers, and utilization of social support (9). In this study, medication safety competence in the older adults includes four dimensions: medication knowledge, medication beliefs, participation in medication decisions, and medication self-management. Within the framework of long-term chronic disease management, medication safety competence serves as a critical link between professional medical care and home-based self-management. It directly influences disease control outcomes, readmission rates, healthcare costs, and patients' quality of life (10).

This competence is particularly crucial for older adults CHD patients who have undergone PCI. This population often requires long-term combination therapy with antiplatelet agents, statins, antihypertensives, and other medications, leading to complex treatment regimens and a high risk of drug interactions (11). Compounded by age-related decline in physiological function, potential cognitive impairment, and frequent multimorbidity, they are more susceptible to medication errors such as incorrect dosing, missed doses, or self-adjusted regimens (12, 13). Studies confirm that inadequate medication safety competence is a significant contributing factor to adverse drug events, treatment discontinuation, and disease recurrence (14). Therefore, systematically assessing and enhancing this competence in this group is not only a core component of implementing secondary prevention for CHD but also a vital pathway toward promoting proactive health management and achieving successful aging.

Currently, most related research assesses patients' medication adherence, self-management, or other related aspects primarily through overall scale scores or single-dimensional measures, treating the study population as a homogeneous group (15, 16). This approach often overlooks the potential structural heterogeneity within the population. Latent Profile Analysis (LPA), as a person-centered statistical method, can identify distinct patient subgroups with different combinations of characteristics based on multiple observed variables, thereby revealing the latent classification structure within a population. Applying LPA allows for a more nuanced delineation of the strengths and weaknesses in medication safety competence across different subgroups, clarifying their unique risk profiles and care needs (17). Consequently, this study aims to employ LPA to develop a latent profile of home medication safety competence among older adults post-PCI patients. The specific objectives are to: (1) identify the potential classes of medication safety competence; (2) describe the characteristic patterns of patients in each class; and (3) explore the demographic, clinical, and psychosocial factors associated with class membership. The findings are expected to provide an empirical basis for identifying high-risk medication management groups, developing stratified and precise medication education strategies, and establishing individualized long-term pharmaceutical care models.

## 2 Method

### 2.1 Participants

This study enrolled older patients who underwent PCI in the cardiology department of a tertiary hospital in Sichuan Province between February and June 2025, using convenience sampling. Inclusion criteria: ① diagnosed with CHD according to established guidelines (18), and having undergone PCI; ② age ≥60 years; ③ duration of PCI-related medication use ≥1 month; ④ provided informed consent and voluntarily participated in the study. Exclusion criteria: ① had severe comorbidities affecting important organs such as the liver, brain, or kidneys; ② severe concurrent cardiac conditions such as heart failure or arrhythmias; ③ history of mental illness or significant cognitive impairment affecting comprehension, expression, memory, or orientation; ④ concurrent participation in another similar research study. Based on Kendall's formula (19), a sample size of 5–10 times the number of independent variables is recommended. This study included 25 independent variables. Accounting for an estimated 20% invalid responses, the minimum required sample was calculated to be 150. The final sample size was 393.

### 2.2 Research tools

#### 2.2.1 General information questionnaire

A self-designed questionnaire was used to collect demographic and disease-related data. Demographic characteristics included gender, age, marital status, education level, place of residence, former occupation, type of medical insurance, average monthly household income per capita, and living arrangements. Disease characteristics comprised disease duration, number of comorbidities, number of medication types, duration of PCI-related medication use, and family reminders for medication.

#### 2.2.2 Medication safety competence scale

Medication safety competence was assessed using the scale developed by Feng et al. (8). This scale was designed to evaluate medication safety competence in patients with chronic diseases. The scale contains 33 items across four dimensions: (1) Medication Knowledge (Items 1–10), assessing understanding of basic drug information, therapeutic purposes, potential adverse reactions, and proper storage; (2) Medication Beliefs (Items 11–17), assessing attitudes toward treatment necessity, medication necessity, and concerns about potential harms; (3) Participation in Medication Decisions (Items 18–24), assessing active involvement in medication-related discussions with healthcare providers, including asking questions and expressing preferences; and (4) Medication Self-Management (Items 25–33), assessing practical abilities such as medication adherence, self-monitoring of drug effects, adverse event recognition, and health self-assessment. Items are rated on a 5-point Likert scale from 1 (completely unaware) to 5 (very aware). Total scores range from 33 to 165, with higher scores indicating stronger medication safety competence. The Cronbach's α in this study was 0.823.

This scale is suitable for LPA because its four dimensions capture distinct yet related aspects of medication safety competence. The 5-point Likert scoring yields sufficient variability to support subgroup identification. Moreover, the scale emphasizes multidimensional competencies, including knowledge, beliefs, decision-making, and self-management behaviors, which aligns well with the person-centered framework of LPA. For the primary analysis, the four dimension mean scores (rather than the 33 individual items) were used as indicators in the LPA to ensure model parsimony and convergence, as detailed in the Section 2.4.

#### 2.2.3 Short-form health literacy scale

Health literacy was assessed using the Short-Form Health Literacy Scale. This scale was originally developed by Chung et al. (35) and translated into Chinese by Li et al. (20). It consists of 10 items across two dimensions: Appraisal of Health Information Literacy and Communication Literacy. A 5-point Likert scale from “Strongly disagree” (1) to “Strongly agree” (5) is used. Higher scores indicate better health literacy. The scale shows robust psychometric properties with a Cronbach's α of 0.948, a split-half reliability of 0.881, and a content validity index of 0.82.

#### 2.2.4 Family APGAR index

The Family APGAR Index, created by Smilkstein (21) and adapted into Chinese by Lyu et al. (22), evaluates family functioning. It consists of five items (adaptation, partnership, growth, affection, and resolve), each scored on a 3-point scale (0 = “hardly ever,” 1 = “sometimes,” 2 = “often”). Total scores classify family function as “severely dysfunctional” (0–3), “moderately dysfunctional” (4–6), or “highly functional” (7–10). Higher scores indicate better family function. In this study, its Cronbach's α was 0.765.

### 2.3 Data collection and quality control

All investigators and three surveyors received uniform training prior to data collection. After obtaining permission from the department head, face-to-face surveys were conducted using paper-based questionnaires. Patients were informed about the study's purpose, significance, and confidentiality, and surveys began only after obtaining their informed consent. Questionnaires were collected and checked for completeness on-site, with invalid ones excluded. For data entry, a double-entry and cross-verification process was implemented to ensure accuracy and enhance the reliability and rigor of the findings.

### 2.4 Statistical analysis

LPA was performed using Mplus version 8.3 (Muthén and Muthén, Los Angeles, CA, USA) software to identify distinct profiles of medication safety competence in older adults post-PCI patients. The mean scores of the four dimensions of the Medication Safety Competence Scale were used as observed indicators for LPA. Models with increasing numbers of latent classes (starting from one) were compared. The optimal model was selected based on a combination of fit indices [Akaike information criterion (AIC), Bayesian information criterion (BIC), adjusted Bayesian information criterion (aBIC), Lo-Mendell-Rubin likelihood ratio test (LMRT), bootstrap likelihood ratio test (BLRT)], entropy values, and the theoretical interpretability of the classes. The resulting classes were then analyzed and named (17). Additional statistical analyses were conducted using IBM SPSS Statistics version 27.0 (IBM Corp., Armonk, NY, United States). Normally distributed continuous data are presented as mean ± standard deviation, with group comparisons made using *ANOVA*. Categorical data are presented as frequencies and percentages, with group comparisons performed using the χ^2^ test or the *Kruskal–Wallis H*-test. For the post-LPA covariate analysis, we used a two-step approach. First, we assigned each participant to a class based on their highest posterior probability. Then, we used these class assignments as known variables in multinomial logistic regression in SPSS. The significance level was set at α = 0.05.

## 3 Results

### 3.1 Sociodemographic characteristics and level of home medication safety competence

A total of 418 questionnaires were collected. We excluded 25 questionnaires because they had incomplete answers or logical errors. The final sample included 393 valid questionnaires (valid response rate = 94.02%). The study included 393 older adults post-PCI patients, with 195 (49.6%) male and 198 (50.4%) female. The mean age was 72.1 ± 7.3. The overall mean score for home medication safety competence was 102.08 ± 16.11. The post-PCI patients included in this study had a duration of PCI-related medication use ranging from 1 month to more than 11 years. This covers different stages from early medication initiation to long-term secondary prevention. Other detailed sociodemographic characteristics are presented in Table 1.

**Table 1:** Univariate analysis of sociodemographic characteristics and associated factors for latent profiles of home medication safety competence in older adults patients after PCI (*n* = 393).

Item [*n* (%)]	Total (*n* = 393)	Low competence group (*n* = 122)	Moderate competence group (*n* = 220)	High competence group (*n* = 51)	*χ^2^/H/F*	*P-*value
Sex						
Male	195 (49.6)	61 (50.0)	104 (47.3)	30 (58.8)	2.220^a^	0.330
Female	198 (50.4)	61 (50.0)	116 (52.7)	21 (41.2)		
Marital status						
Married	297 (75.6)	88 (72.1)	163 (74.1)	46 (90.2)	6.952^a^	0.031
Unmarried/ divorced/widowed	96 (24.4)	34 (27.9)	57 (25.9)	5 (9.8)		
Educational level						
Illiterate	49 (12.5)	21 (17.2)	24 (10.9)	4 (7.8)	45.606^c^	< 0.001
Primary school	171 (43.5)	67 (54.9)	90 (40.9)	14 (27.5)		
Junior high school	110 (28.0)	25 (20.5)	74 (33.6)	11 (21.6)		
High school or above	63 (16.0)	9 (7.4)	32 (14.5)	22 (43.1)		
Residence						
Urban	197 (50.1)	47 (38.5)	115 (52.3)	35 (68.6)	13.957^a^	< 0.001
Rural	196 (49.9)	75 (61.5)	105 (47.7)	16 (31.4)		
Number of comorbidities						
≤ 1	88 (22.4)	24 (19.7)	52 (23.6)	12 (23.5)	2.955^b^	0.565
2–3	182 (46.3)	60 (49.2)	103 (46.8)	19 (37.3)		
≥4	123 (31.3)	38 (31.1)	65 (29.5)	20 (39.2)		
Duration of PCI-related medication use (years)						
≤ 5	133 (33.8)	40 (32.8)	82 (37.3)	11 (21.6)	9.635^b^	0.043
6–10	126 (32.1)	38 (31.1)	74 (33.6)	14 (27.5)		
≥11	134 (34.1)	44 (36.1)	64 (29.1)	26 (51.0)		
Number of medication types						
≤ 2	103 (26.2)	27 (22.1)	57 (25.9)	19 (37.3)	7.088^b^	0.131
3–5	143 (36.4)	48 (39.3)	84 (38.2)	11 (21.6)		
≥6	147 (37.4)	47 (38.5)	79 (35.9)	21 (41.2)		
Family member working in healthcare						
No	316 (80.4)	110 (90.2)	173 (78.6)	33 (64.7)	15.791^a^	< 0.001
Yes	77 (19.6)	12 (9.8)	47 (21.4)	18 (35.3)		
Frequency of family medication reminders						
Never	68 (17.3)	49 (40.2)	18 (8.2)	1 (2.0)	125.320^c^	< 0.001
Occasionally	257 (65.4)	70 (57.4)	166 (75.5)	21 (41.2)		
Frequently	68 (17.3)	3 (2.5)	36 (16.4)	29 (56.9)		
Age (years)	72.1 ± 7.3	73.4 ± 6.9	71.9 ± 7.4	69.9 ± 7.5	4.105^d^	0.017
Health literacy score	33.6 ± 5.1	31.7 ± 4.9	33.5 ± 4.6	38.5 ± 4.2	39.001^d^	< 0.001
Family APGAR score	6.1 ± 1.8	5.8 ± 2.0	6.0 ± 1.6	7.4 ± 1.6	15.395^d^	< 0.001

^a^Chi-square test value.

^b^H-value (Kruskal–Wallis H-test).

^c^Fisher's exact test value.

^d^F-value (ANOVA).

### 3.2 LPA of home medication safety competence

**Figure 1:**
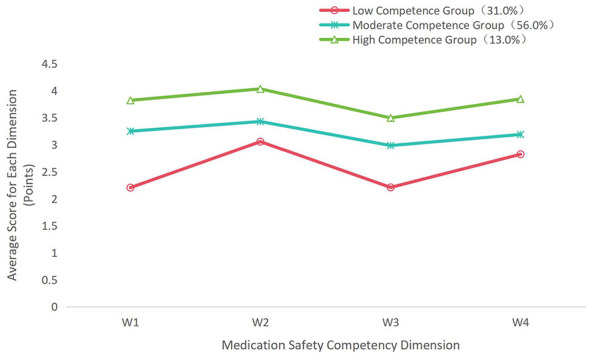
Characteristics distribution of latent profiles in home medication safety competence among older adults patients after PCI. W1 = medication knowledge; W2 = medication beliefs; W3 = participation in medication decisions; W4 = medication self-management. The three lines in the figure represent the three latent classes: low competence group (31.0%), moderate competence group (55.9%), and high competence group (12.9%). The scores of the three classes go up in parallel across the four dimensions.

Using the average scores of the four dimensions of the Medication Safety Competence Scale as observed indicators, LPA models with one to four latent profiles were fitted sequentially (Table 2). As the number of profiles increased, the values of AIC, BIC, and aBIC gradually decreased, all entropy values exceeded 0.8, and both the LMRT and BLRT were statistically significant (P < 0.001). The rate of decrease in the information criteria diminished notably when the number of profiles increased from two to three. The 3-profile model also yielded the highest entropy value (0.875). The P-value for the 4-profile model was 0.1059 (>0.05). This means the 4-profile model was NOT significantly better than the 3-profile model. Therefore, the 3-profile model was selected as the best-fitting solution. A latent profile plot based on this classification is presented in Figure 1. The scores of the three classes show a consistent “low, middle, high” increasing trend across the four dimensions. The lines are nearly parallel. This suggests that the classes mainly differ in their level of competence, not in the shape of their patterns. Based on the score patterns across dimensions, the three classes were named as follows: Class 1 (low competence group, n = 122, 31.0%): patients in this group scored low across all dimensions of medication safety competence. Class 2 (moderate competence group, n = 220, 55.9%): patients in this group demonstrated moderate scores across all dimensions. Class 3 (high competence group, n = 51, 13.0%): patients in this group achieved high scores across all dimensions.

**Table 2:** Model fit indices for LPA of home medication safety competence in older adults patients after PCI (*n* = 393).

Model	AIC	BIC	aBIC	Entropy	LMRT (*P*)	BLRT (*P*)	Class probabilities
1	9,348.778	9,380.568	9,355.184	—	—	—	—
2	8,968.483	9,020.143	8,978.894	0.802	0.0031	< 0.001	0.392/0.608
**3**	**8,837.834**	**8,909.362**	**8,852.249**	**0.875**	**0.0071**	**< 0.001**	**0.560/0.310/0.130**
4	8,788.234	8,879.631	8,806.653	0.844	0.1059	< 0.001	0.298/0.287/0.364/0.051

AIC, Akaike information criterion; BIC, Bayesian information criterion; aBIC, adjusted Bayesian information criterion; LMRT, Lo-Mendell-Rubin likelihood ratio test; BLRT, bootstrap likelihood ratio test. See Abbreviations section for full definitions. The 3-profile model was selected as the optimal solution (bolded).

### 3.3 Univariate analysis of the factors associated with latent profiles of home medication safety competence

The analysis revealed statistically significant differences among the three patient profiles (*P* < 0.05) concerning age, marital status, educational level, place of residence, duration of medication use, whether family members work in the healthcare sector, and the frequency of family reminders for medication. Furthermore, significant differences (*P* < 0.001) were observed in Health Literacy scores (31.7 ± 4.9, 33.5 ± 4.6, and 38.5 ± 4.2) and Family APGAR scores (5.8 ± 2.0, 6.0 ± 1.6, and 7.4 ± 1.6) across the three groups. Details are presented in Table 1.

### 3.4 Multivariate analysis of the factors associated with latent profiles of home medication safety competence

Using the three latent profiles as the dependent variable, a multinomial logistic regression was performed with all variables that showed statistical significance in the univariate analysis as independent variables. The assignment of categorical variables is shown in Table 3, while age, health literacy, and family APGAR scores were entered as continuous variables. The results indicated that age, marital status, educational level, frequency of family medication reminders, health literacy, and family support were significant factors associated with home medication safety competence in older adults post-PCI patients (all *P* < 0.05). Details are presented in Table 4.

**Table 3:** The way of assigning values to the independent variables.

Variables	Assignment method
Marital status	Married = 0; unmarried/divorced/widowed = 1
Educational level	Illiterate = (1, 0, 0); primary school = (0, 1, 0); junior high school = (0, 0, 1); high school or above = (0, 0, 0)
Residence	Urban = 0; rural = 1
Duration of PCI-related medication use (years)	≤ 5 = 0; 6–10 = 1; ≥11 = 2
Family member working in healthcare	No = 0; yes = 1
Frequency of family medication reminders	Never = 0; occasionally = 1; frequently = 2
Age (years)	Raw value input
Health literacy score	Raw value input
Family APGAR score	Raw value input

**Table 4:** Multinomial logistic regression analysis of factors associated with latent profiles of home medication safety competence in older adults patients after PCI (*n* = 393).

Variable	β	SE	Wald *χ^2^*	*P-*value	OR	95% CI
Comparison: C1 vs. C2^a^						
Constant	−0.757	1.878	0.163	0.687	—	—
Frequency of family medication reminders						
Never	1.167	0.393	8.798	0.003	3.213	1.486–6.947
Occasionally	1.241	0.298	17.299	< 0.001	3.458	1.927–6.206
Family APGAR score	0.076	0.026	8.223	0.004	1.079	1.024–1.136
Comparison: C1 vs. C3^a^						
Constant	−9.599	3.491	7.561	0.006	—	—
Educational level						
Primary school	−2.439	0.657	13.803	< 0.001	0.087	0.024–0.316
Junior high school	−2.016	0.698	8.339	0.004	0.133	0.034–0.523
Frequency of family medication reminders						
Never	−2.395	0.688	12.101	0.001	0.091	0.024–0.352
Occasionally	−1.123	0.531	4.471	0.034	0.325	0.115–0.921
Age (years)	−0.081	0.033	5.987	0.014	0.922	0.864–0.984
Health literacy score	0.372	0.061	36.955	< 0.001	1.451	1.287–1.635
Family APGAR score	0.486	0.156	9.665	0.002	1.627	1.197–2.21
Comparison: C2 vs. C3^b^						
Constant	8.841	3.268	7.321	0.007	—	—
Marital status						
Married	−1.31	0.621	4.458	0.035	0.27	0.08–0.91
Educational level						
Primary school	1.673	0.557	9.011	0.003	5.329	1.787–15.889
Junior high school	2.029	0.589	11.88	0.001	7.606	2.399–24.112
Frequency of family medication reminders						
Occasionally	1.228	0.502	5.974	0.015	3.414	1.275–9.14
Age (years)	0.063	0.031	4.271	0.039	1.065	1.003–1.132
Health literacy score	−0.296	0.058	25.968	< 0.001	0.744	0.664–0.833
Family APGAR score	−0.476	0.15	10.016	0.002	0.621	0.463–0.834

C1 = low competence group; C2 = moderate competence group; C3 = high competence group.

OR, odds ratio; CI, confidence interval.

Data are presented as β coefficient, standard error (SE), Wald chi-square (Wald χ^2^), P-value, OR, and 95% CI. OR = exp (β).

^a^Reference group is C1.

^b^Reference group is C3.

## 4 Discussion

### 4.1 Heterogeneity exists in home medication safety competence among older adults patients post-PCI

This study identified three distinct latent profiles of home medication safety competence among older adults post-PCI patients. The three classes mainly differ in their level of competence. The Low competenc group accounted for 31% of the sample. Patients in this profile were older (73.4 ± 6.9 years), predominantly had an elementary school education or less, were more likely to reside in rural areas, and had lower scores in family support and health literacy. Family members “never” reminded them to take medication. Collectively, these demographic, educational, environmental, and support-related disadvantages are associated with lower ability to develop effective self-management skills for medication safety (23), fostering a reliance on passive guidance from healthcare professionals rather than active, self-directed behavior. This group should be a primary focus for nursing interventions. Recommended strategies include the use of simple, visual medication instructions (such as pictures, large print, and color coding), the involvement of family members as co-managers of medication, and the provision of regular follow-up through simplified educational materials and enhanced family support. The Moderate competence group comprised the majority (55.9%). These patients were slightly younger (71.9 ± 7.4 years), mostly had a middle school education, exhibited moderate levels of family support and health literacy, and received “occasional” family reminders. While they possess basic health knowledge and some family support, enabling a degree of cooperation with treatment, there remains room for improvement in proactive medication management, adverse effect recognition, and sustained adherence. Nursing efforts for this group should focus on strengthening self-monitoring and consistent management skills (37). Recommended strategies include teaching patients to monitor their own health (e.g., tracking blood pressure and recognizing side effects), using medication reminders (such as pillboxes, phone alarms, or medication apps), encouraging patients to ask questions during clinic visits, and providing brief education on potential drug interactions. The High competence group was the smallest (12.9%). These patients were the youngest (69.9 ± 7.5 years), predominantly had a high school education or above, mostly lived in urban areas, received “frequent” family reminders, and had the highest scores in health literacy and family support. Higher education and robust family support provide cognitive and social resources that empower them to actively seek medication information and adhere to treatment plans (24). As a result, this group could be leveraged as peer educators in population-level health interventions. Recommended strategies include involving these patients as peer educators for others, offering advanced information on complex medication management, and sustaining their competence through regular reinforcement and updates.

### 4.2 Deficiencies in the family support system are significantly associated with low home medication safety competence among older adults patients post-PCI

Our findings show that being married, receiving frequent family medication reminders, and having higher family support significantly increased the likelihood of belonging to the Moderate or High competence groups compared to the Low competence group. This underscores that strong family support is crucial not only for adherence but also for fostering proactive medication management, aligning with prior research (25). A spouse, as the primary caregiver, provides irreplaceable emotional companionship, behavioral prompting, and shared health decision-making (26). This consistent, personalized support enhances patients' sense of agency, facilitating a shift from passive dependency to self-directed management (27). The frequency of family medication reminders was particularly impactful. Compared to “frequent reminders” (reference), “never” receiving reminders increased the odds of being in the Low competence group vs. the Moderate competence group (C1 vs. C2) by approximately 3.2-fold (OR = 3.213, 95% CI: 1.486–6.947). When comparing the Low competence group to the High competence group (C1 vs. C3), “never” receiving reminders was associated with even lower odds of being in the high competence group (OR = 0.091, 95% CI: 0.024–0.352), indicating a substantial disadvantage. For older adults with natural cognitive decline, regular external cues are a vital safety mechanism. The absence of such prompts increases the risk of errors, trapping patients in a state of low competence (38). Higher family APGAR scores were also associated with a lower probability of being in the Low competence group; for example, in the comparison between Low and High competence groups (C1 vs. C3), each one-point increase in the Family APGAR score was associated with higher odds of being in the High competence group (OR = 1.627, 95% CI: 1.197–2.21). Family support extends beyond reminders to include emotional bonding and shared learning. A lack of such holistic support can foster isolation and helplessness, undermining the motivation for active health management (28). Interventions must therefore build “co-management partnerships,” combining external support with strategies to activate patient participation.

### 4.3 Low educational attainment and advanced age are important individual factors that hinder older adults patients post-PCI from achieving high-level self-medication management competency at home

Compared to the High competence group (C3), patients with an education level of junior high school or below and those who were older had a significantly higher probability of belonging to the Low competence group (C1). Specifically, compared to those with higher education (reference), having primary school education was associated with lower odds of being in the High competence group vs. the Low competence group (OR = 0.087, 95% CI: 0.024–0.316 in C1 vs. C3), as was junior high school education (OR = 0.133, 95% CI: 0.034–0.523). Additionally, for each 1-year increase in age, the odds of belonging to the high competence group (vs. low competence) decreased by approximately 7.8% (OR = 0.922, 95% CI: 0.864–0.984). This suggests that lower education and advanced age are key individual barriers to achieving high-level self-management, consistent with other studies (29). An education at or below junior high school may limit a patient's ability to understand, process, and apply complex medication information (40). Given the intricate, multi-drug regimens post-PCI, this constraint can hinder accurate risk identification and proper administration, preventing the development of effective self-management skills (39). Furthermore, advanced age often brings cognitive decline and multimorbidity, compounding the challenges of safe medication management (30). Interventions for this subgroup should employ simple, visual, and intuitive medication aids.

### 4.4 The higher the health literacy score, the greater the home medication safety competence

Our findings reveal that higher health literacy significantly increases the likelihood of older adults patients post-PCI belonging to the high competence profile (OR = 1.451, 95% CI: 1.287–1.635 in C1 vs. C3), consistent with prior research (31). This may be explained by patients' enhanced capacity for proactive knowledge acquisition and risk identification, which are foundational to autonomous medication management (32). Concurrently, enhanced family support was also significantly associated with a greater probability of belonging to the High competence group (OR = 1.627, 95% CI: 1.197–2.21 in C1 vs. C3), which corroborates existing research (36). The underlying mechanism may be that robust family support creates a favorable environmental context that facilitates self-directed medication management (33). These results suggest a dual-pathway model where individual cognitive capacity (health literacy) and environmental contextual support (family support) interact synergistically. Health literacy provides the essential cognitive foundation for self-management, while family support offers crucial emotional and behavioral reinforcement. Notably, even when compared to the Moderate competence group (C2 vs. C3), both health literacy (OR = 0.744, 95% CI: 0.664–0.833) and family support (OR = 0.621, 95% CI: 0.463–0.834) remained significant predictors (34). This underscores that medication safety competence exists on a dynamic continuum. Improvement requires ongoing, concurrent strengthening of both internal capabilities and external support systems. Consequently, intervention strategies must be comprehensive, integrating patient-level education to build health literacy with family-level engagement to optimize the support environment, thereby fostering sustainable development of medication safety competence.

## 5 Limitations

This study has several limitations. First, the use of convenience sampling from a single tertiary hospital in Sichuan Province may limit the representativeness of the sample. Caution should be exercised when generalizing the findings to broader populations, other regions, or primary care settings. Future research should involve multi-center studies with larger samples to enhance generalizability. Second, as a cross-sectional design, this study cannot establish causal relationships among health literacy, family support, and medication safety competence, nor can it capture their dynamic changes over time. Subsequent studies could adopt longitudinal or interventional designs to further explore causal pathways and developmental trajectories. Third, while strict inclusion and exclusion criteria were applied to control for obvious cognitive impairment, the lack of standardized cognitive screening for all participants means that undetected mild cognitive impairments may have acted as confounding variables. Future studies are recommended to incorporate systematic cognitive and psychosocial assessments at baseline to better control for potential biases. Fourth, data collection relied primarily on self-reported questionnaires. Although validated instruments were used, responses may still be subject to social desirability or recall bias. Objective measures such as pharmacy records, electronic monitoring devices, or caregiver reports could be incorporated in the future for multi-source verification. Fifth, for the post-LPA covariate analysis, we used a two-step approach. First, we assigned each participant to a class based on their highest posterior probability. Then, we used these class assignments as known variables in multinomial logistic regression in SPSS. It is important to note that this method does not account for the uncertainty in class classification. This may lead to smaller standard errors and a higher risk of false positive results. Future studies should use a three-step method (such as R3STEP or BCH) to obtain more robust estimates.

## 6 Conclusion

Using LPA, this study revealed significant heterogeneity in home medication safety competence among older adults post-PCI patients. Competence is associated with a combination of factors, including educational level, age, health literacy, and family support. Clinical nursing practice should adopt stratified and precise intervention strategies tailored to the characteristics of different competence profiles, with particular emphasis on boosting health literacy and fortifying family support systems.

## Data Availability

The raw data supporting the conclusions of this article will be made available by the authors, without undue reservation.
